# Outcomes of antiretroviral treatment program in Ethiopia: Retention of patients in care is a major challenge and varies across health facilities

**DOI:** 10.1186/1472-6963-11-81

**Published:** 2011-04-18

**Authors:** Yibeltal Assefa, Abiyou Kiflie, Dessalegn Tesfaye, Damen Haile Mariam, Helmut Kloos, Wouters Edwin, Marie Laga, Wim Van Damme

**Affiliations:** 1 Medical Services Directorate, Federal Ministry of Health, Addis Ababa, Ethiopia; 2 School of Public Health, Addis Ababa University, Addis Ababa, Ethiopia; 3 Department of Epidemiology and Biostatistics, University of California, San Francisco, USA; 4 Department of Sociology, University of Antwerp, Antwerp, Belgium; 5 Department of Microbiology, Institute of Tropical Medicine, Antwerp, Belgium; 6 Department of Public Health, Institute of Tropical Medicine, Antwerp, Belgium

## Abstract

**Background:**

Many resource-limited countries are scaling up antiretroviral treatment (ART) towards universal access. However, there are few studies which evaluated outcomes of ART programs in these countries. In addition, these studies generally include a limited number of facilities and patients creating a clear need for studies with a wide range of facilities and large numbers of patients. In this study, we intended to evaluate the outcomes of the ART services in 55 health facilities in Ethiopia.

**Methods:**

A retrospective longitudinal study was conducted to determine levels of patient retention in care, CD4 count and shift to second-line ART regimen in 30 hospitals and 25 health centers selected as sentinel sites for monitoring the outcomes of ART program in the country. The outcomes were determined at baseline, after 6, 12 and 24 months on ART. Data was collected from routine patient registers and charts, and entered and analyzed using EPI-Info statistical software.

**Results:**

Health facilities were able to retain 29,893 (80%), 20,079 (74%) and 5,069 (68%) of their patients after 6, 12 and 24 months on ART, respectively. Retention rates vary across health facilities, ranging from 51% to 85% after 24 months on ART. Mortality was 5%, 6% and 8% after 6, 12 and 24 months on ART. More than 79% of patients with available CD4-cell counts had a baseline CD4-cell counts less than 200 cells per micro-liter of blood. The median CD4-cell counts (based on patients who were retained after 24 months on ART) increased from 125 (inter-quartile (IQ), 68-189) at baseline to 242 (IQ, 161-343), 269 (IQ, 185-380) and 316 (IQ, 226-445) cells per micro-liter after 6, 12, and 24 months on ART, respectively. The transition to second-line ART remained very low, 0.33%, 0.58% and 2.13% after 6, 12 and 24 months on ART.

**Conclusion:**

The outcomes of the ART services in the 55 health facilities in Ethiopia are similar to those in other countries. Retention of patients in care is a major challenge and varies across health facilities with high, medium and low retention rates. We therefore recommend further studies to understand the organization of care in health facilities with high, medium and low retention rates. It is also imperative that early initiation of patients on ART is taken seriously as more than 79% of the patients had baseline CD4-cell counts less than 200 cells per micro-liter of blood. Finally, we recommend that the shift to second-line ART might be too low and warrants close monitoring.

## Background

The use of antiretroviral treatment (ART) has substantially decreased morbidity and mortality in people living with HIV/AIDS (PLwHAs) [1-3]. As a result, there have been several initiatives for the rapid scale-up of ART in resource-limited countries (RLCs). Some of these initiatives are: (i) increased funding [4]; (ii) a dramatic reduction in prices of antiretroviral medicines [5]; and (iii) the public health approach to ART delivery [6].

Experiences on ART scale-up using the public health approach have been reported in many RLCs, including Ethiopia; and these reports indicated that it is possible to scale up ART in RLCs [5,7-16].

However, the few studies which evaluated the outcomes of large scale national ART programs in RLCs [10,12,16] generally only included a limited number of health facilities and patients. As a result, it will be difficult to generalize the findings to other settings. Hence, there is a need for large-scale studies, which are conducted in a wide range of health facilities and large numbers of patients, to evaluate the outcomes of ART programs.

The objective of this study was to evaluate the outcomes of the rapidly expanding ART program in Ethiopia, which aims to provide universal access to ART, using the commonly measured outcome indicators of ART programs: mortality, loss to follow-up, retention in care, change in CD4-cell counts and shift to second-line ART [17].

## Methods

### The antiretroviral treatment program in Ethiopia

According to the most recent estimates, about 1 million people (2.3% of the adult population) were living with HIV in Ethiopia in 2009. In the same year, more than 300, 000 people needed ART [18]. A fee-based ART program was officially started in 2003. Subsequently, a number of initiatives have been undertaken to expand the availability of ART in Ethiopia, including those by the Global Fund to Fight AIDS, Tuberculosis and Malaria, the US President's Emergency Plan For AIDS Relief (PEPFAR), the Ethiopian North American Health Professionals Association, the Clinton Foundation and the Ethiopian Red Cross Society [19]. As a result, a free ART program was launched in early 2005. Subsequently, ART services have been decentralized and made available at increasing numbers of both health centers and hospitals since August 2006 [20].

The number of patients who were ever started on ART in Ethiopia increased from 900 at the beginning of 2005 to more than 180,000 by the end of 2008, and the number of patients who were enrolled for ART increased from 2,700 to 5,000 per month. The proportion of patients receiving ART outside Addis Ababa increased from 35% in 2005 to 75% in 2008 [7,8], as a result of the free and decentralized ART program in the country [20]. The proportion of patients who received ART in health centers increased almost 10-fold between October 2006 and October 2007, from 1.1% to 11.4% [7].

### Study design

A retrospective longitudinal study was conducted to assess the outcomes of the ART services in hospitals and health centers from all regions and administrative cities in the country. The study included health facilities from both public and private sectors.

A list of all health facilities that were providing ART services in all regions of the country (367 health facilities) was used as a sampling frame. Fifty-five health facilities (30 hospitals and 25 health centers) which were providing ART for more than 200 patients by November 2008, the time the study was initiated, were included in the study. Health facilities providing services for less than 200 patients were excluded from the study because the service delivery was relatively not well matured, and outcomes of the services were assumed to be affected by lack of experience. Hence, it was decided to include health facilities with more than 200 patients on ART. These 55 health facilities were not randomly selected. They were selected in such a way that at least 15% of health facilities in all regions in the country were included, and all levels of health facilities (tertiary and secondary hospitals and health centers) were represented. These facilities will be used as sentinel sites to monitor the outcomes of ART program in the country. Cohort database for ART patient monitoring was introduced into the national ART program in September 2006. Hence, the study included patients who started ART between September 2006 and August 2008. The patient monitoring for the study ended in March 2009, with the minimum cohort period of 6 months and the maximum of24 months. Thus, the total study period was 30 months.

Systematic sampling technique was used to select individual patient charts and to identify their CD4-cell counts. Two hundred patient charts were selected from health facilities with more than 1000 patients on ART, and 150 patient charts from health facilities with 200-1000 patients on ART. These sample sizes for number of charts were determined based on the recommendation of WHO's Annual Patient Monitoring [17]. The charts of patients who were retained after 24 months on ART, and had their CD4 -cell counts determined at baseline, 6, 12 and 24 months on ART were used to examine the trend in the CD4-cell counts.

### Antiretroviral treatment regimen and patient follow-up

The first-line ART regimen used consists of two nucleoside reverse transcriptase inhibitors plus one non-nucleoside reverse transcriptase inhibitor. Stavudine-lamivudine-nevirapine accounted for 46%, zidovudine-lamivudine-nevirapine for 18%, stavudine-lamivudine-efavirenz for 23%, and zidovudine-lamivudine-efavirenz for 13% of the patients taking ART in the country during the study period. The second-line regimen consists of 2 nucleoside reverse transcriptase inhibitors plus one protease inhibitor (lopinavir/ritonavir): abacabir-didanosine-lopinavir/ritonavir for 24% or tenofovir-didanosine-lopinavir/ritonavir for 76% of the patients [21].

Patients were started on ART based on clinical and immunological criteria: World Health Organization stage 4; stage 3 and CD4-cell count less than 350; or CD4-cell count less than 200 cells per micro-liter of blood [21]. Patients had their chemistry, hematology and CD4-cell counts determined before ART initiation and every 6 months afterwards. Patients might have their CD4-cell counts determined whenever necessary. However, the CD4-cell counts were recorded in the register for those at baseline, 6, 12 and 24 months on ART. The time margin to document the CD4-cell counts was one week before and after the date of appointment for the routine CD4-cell counts at baseline, 6, 12 and 24 months on ART. Patients who were eligible for ART would pass through both group and individual counseling sessions for ART adherence, and would then be appointed for ART initiation after 2 weeks. They were also screened for active opportunistic infections (OIs), such as tuberculosis; and patients with active OIs were treated according to the national guidelines [21].

All patients who received nevirapine-based ART were scheduled to return 2 weeks after ART initiation for review and dose escalation, unless contraindicated. Once patients were started on ART, they were scheduled to have visits every 2 weeks for the first month, every month for the first 3 months, and every 3 months afterward, unless they encountered problems [21].

### Data collection

Data were collected from patient registers, a hybrid of electronic and paper-based patient management systems, on mortality, loss to follow-up, transfer in and out, functional status and regimen change to second-line treatment. Patients die either at health facilities or in the community. Mortality data for patients who died at health facilities were collected from patient charts and registers; and, for patients who died in the community mortality data was collected through home visits and telephone call of family members. Clinical charts were used to collect the data on CD4-cell counts. The data on CD4-cell counts were entered into a separate database for analysis, and the median and inter-quartile (IQ) CD4-cell counts were determined.

### Measurements and statistical analysis

Mortality, loss to follow-up, retention, functional status, median CD4-cell counts and shift to second-line ART regimen were primary outcomes for this study and were determined at baseline, after 6, 12, and 24 months on ART. Data were entered, coded, cleaned and analyzed using EPI-Info 2002 statistical software.

### Ethics

Federal HIV/AIDS Prevention and Control Office of Ethiopia coordinates the HIV/AIDS response in the country. The Office has got an Institutional Review Committee which reviews operational studies related to HIV/AIDS. Our research proposal was reviewed by the committee, and we got an approval to conduct the study.

## Results

We found that 37,466 patients were started on ART at least 6 months prior to the censoring date. During this 6- month cohort, 2,304 (6%) patients were transferred out to other health facilities, 125 (0.33%) patients were shifted to second-line ART, 1,897 (5%) patients died, 5,676 (15%) patients were lost to follow-up, and 29,893 (80%) patients were alive and on ART (Table 1).

**Table 1:** Outcomes of patients on ART in 55 health facilities in Ethiopia between September 2006 and August 2008

Duration of cohort	Patients in the cohort (a)	Transferred in during the cohort (b)*	Transferred out during the cohort (c)	Active cohort (d = a+b-c)	Second-line ART during the cohort ((e) = e/d)(%)**	Died during the cohort ((f) = f/d) (%)	Loss to follow-up during the cohort ((g) = g/d) (%)	Alive and on ART at the end of the cohort (h)
6 months	38,061	1,709	2,304 (6%)	37,466	125 (0.33%)	1,897 (5%)	5,676 (15%)	29,893 (80%)
12 months	27,668	1,904	2,511 (9%)	27,061	158 (0.58%)	1,716 (6%)	5,266 (19%)	20,079 (74%)
24 months	7,519	814	882 (12%)	7,451	159 (2.13%)	619 (8%)	1,763 (24%)	5,069 (68%)

*The duration on ART of patients transferred in is calculated from the date they started ART irrespective of where they started the treatment.

**Column e is not mutually exclusive from columns f, g and h; while columns f, g and h are mutually exclusive. Patients who shifted to second-line ART during the 6 months cohort were counted as patients on second-line ART in the 12 and 24 months cohort if they were alive and on ART after 12 and 24 months on ART. Moreover, patients on second-line ART during the 12 months cohort were also counted as patients on second-line ART in the 24 months cohort if they were alive and on ART after 24 months on ART.

Of the 27,061 patients who were followed for 12 months. 2,511 (9%) were transferred out to other facilities, 158 (0.58%) were shifted to second-line ART, 1,716 (6%) died, 5,266 (20%) were lost to follow-up, and 20,079 (74%) were alive and on ART (Table 1).

Out of the 7,451 patients who were in the 24-months cohort, 882 (12%) were transferred out to other facilities, 159 (2.13%) were shifted to second-line ART, 619 (8%) died, 1,763 (24%) were lost to follow-up, and 5,069 (68%) were alive and on ART (Table 1).

We also found that the proportion of patients who shifted to second line treatment remained very low and progressed slowly, from 0.33% after 6 months on ART to 0.58% and 2.13% after 12 and 24 months on ART, respectively (Table 1).

**Figure 1:**
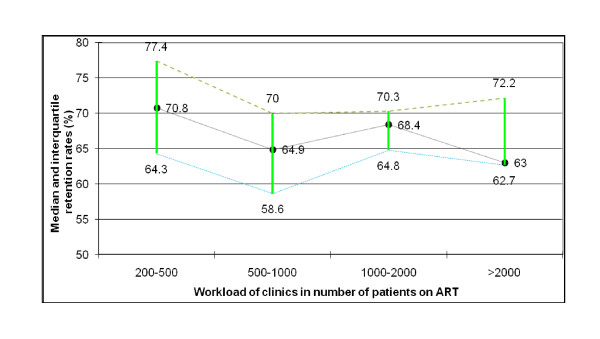
**Retention rates of patients after 24 months on ART**.

We found that retention rates varied considerably among health facilities, ranging from 51% to 85% after 24 months on ART. We also found that the retention rates after 24 months on ART in health facilities with 200-500 (34 health facilities), 500-1000 (10 health facilities), 1000-2000 (7 health facilities) and greater than 2000 (4 health facilities) patients ever started on ART are not different (Figure 1). There was also no difference in retention rates in ART clinics in big urban cities (with median (IQ) retention rates of 70.8 (64.3-74.5)) and rural areas (with median (IQ) retention rates of 67.2 (62.7-74.4)). We also found that there was no difference in retaining patients in care in the different geographical zones (North, Central, South, West and East) of the country where different implementing partners are supporting the ART delivery.

**Figure 2:**
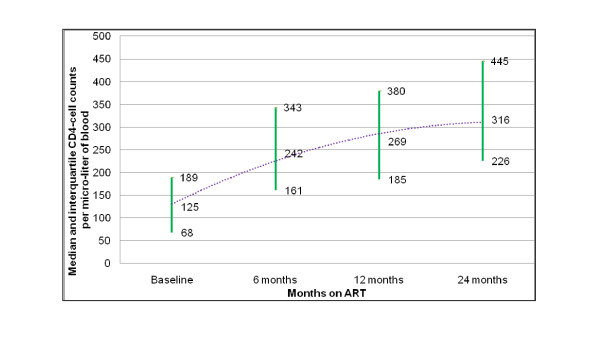
**Trend of median CD4-cell counts of 4,410 patients on ART in 55 health facilities in Ethiopia, 2006-2008**.

The trend in median CD4-cell counts was drawn based on patients retained after 24 months on ART (5,069 patients) and had their CD4-cell counts determined at baseline, 6, 12 and 24 months on ART (4,410 patients (87%)). Accordingly, the median CD4-cell counts was 125 (IQ, 68-189) cells per micro-liter at baseline, and it increased to 242 (IQ, 161-343), 269 (IQ, 185-380) and 316 (IQ, 226-445) cells per micro-liter after 6, 12 and 24 months on ART, respectively (Figure 2). Total CD4-cell count rose by 193 cells during the 24 months of treatment, out of which 61% (117 cells per micro-liter) was achieved during the first 6 months of treatment, compared to only 14% (27 cells) during the second 6 months of the first year of treatment and only 25% (47 cells) during the second year of treatment. The proportion of patients with CD4-cell count above 200 cells per micro-liter was 21% at baseline, and it increased to 63%, 70% and 81% after 6, 12 and 24 months on ART, respectively.

The proportion of patients that had a "working functional status" at baseline was 60%, and this increased to 84%, 86% and 88% after 6, 12 and 24 months on ART, respectively, of those still retained in care.

## Discussion

This study showed that health facilities in Ethiopia were able to retain 80%, 74% and 68% of their patients after 6, 12 and 24 months on ART, respectively. Attrition from care and treatment sites was mainly due to loss to follow-up, and was much higher during the 6-month cohort than the other cohorts (Table 1). We found that retention rates are variable among health facilities; but, it does not vary with the size of the ART clinic or patient load in the health facility (Figure 1). There were improvements in CD4-cell counts (Figure 2) and functional status of patients during the course of their treatment, with enormous improvements occurring during the first 6 months of treatment. The study also showed that the transition to second-line treatment remained very low and progressed slowly, from 0.33% at sixth month to 2.13% after 24 month on ART.

Mortality and loss to follow-up rates after 6 (5% and 15%), 12 (6% and 20%) and 24 (8% and 24%) months on ART were comparable to those in other developing countries such as Malawi, Haiti and China [22-24]. The high mortality and loss to follow-up in the 6-month cohort in our study is similar to the findings in many other studies which show that loss to follow-up and death mostly occur during the first 6 months of treatment [25-30]. Studies in Zambia and Uganda found similarly high rates of loss to follow-up, 21% and 24%, respectively, in the first year of ART [16,31]. This is an indication that health facilities optimize adherence and close patient monitoring in early months of treatment for long-term clinical, immunological and virological benefits for their patients.

Our study strengthens the argument that evaluations of ART programs in RLCs should consider not only the number of new patients started on ART, but also the number of patients retained in long-term care [30]. It is evident that patients visiting health facilities more frequently have a greater increase in the median CD4-cell count, a greater drop in the median viral load and a better chance of long-term survival [32-34]. We think recognizing and addressing the challenges of retaining patients in care potentially contribute a lot to improving the long-term impact of ART programs. Hence, it is imperative that ART programs set up a system that ensures regular follow-up of patients to care and treatment services.

ART programs can develop various strategies to help ensure patient retention in long-term care. One possible strategy is that programs utilize different eligibility criteria to restrict provision of treatment only to patients thought to be retained in long-term care. However, such selection process is undesirable in public sector programs that envision universal access to ART. Identifying and addressing the major impediments to retention may be more appropriate than excluding patients with such impediments. Another common measure to ensure patient retention in HIV treatment programs involves tracing patients who have missed an appointment [35]. However, given the large number of patients and limited financial and human resources available in RLCs, it will be more cost-effective to prevent loss to follow-up than to track patients who have missed visits [35].

Treatment, care and support programs can also be designed to provide holistic services that put emphasis on continuation of care for patients. Programs that provide comprehensive clinical and non-clinical services can help to address the social, economical and contextual barriers which hinder regular follow-up. In general, we believe that identifying the barriers and facilitators for retention in care, and capitalizing on the facilitators and addressing the barriers can significantly increase the coverage and effectiveness of ART programs in the long-term.

We also looked at the shift to second-line ART in 30 hospitals which are qualified to provide these services. We found that the shift to second-line ART was 0.33%, 0.58% and 2.13% after 6, 12 and 24 months on ART, respectively. These rates are comparable to those in other resource-limited settings [36], but much lower than in China (12% and 22% after 12 and 24 months on ART, respectively) [37]. We, thus, think that the shift to second-line ART needs close monitoring as it may have effect on patient outcomes.

This study has both strengths and weaknesses. One of the strengths is that the cohort was large (55 health facilities and 37, 466 patients on ART) and that all regions of the country were well represented. Hence, the findings from the study can be generalized to other health facilities in the country. The second strength of the study is that it included many outcome indicators: mortality, loss to follow-up, retention, transfer out, CD4-cell count, functional status, and shift to second-line treatment.

The first weakness of the study is that loss to follow-up and transfer out rates are high, and as such, the outcome for these patients is not known. It is possible that patients who were lost to follow-up might have died, in which case the survival benefit would have been over-estimated. For patients who were transferred to another treatment site, it could be argued that their outcomes would be similar to those who remained in the same facility where they started treatment; however, it might not be the case. The second weakness of the study is that the data for CD4-cell counts were not complete either because they were not determined, or if determined, not registered. In spite of these weaknesses, this study provides good insights into the performance of the Ethiopian national ART program highlighting that retention of patients in care in Ethiopia is comparable to that of other RLCs, remains a major challenge and varies across health facilities.

## Conclusion

The ART program in Ethiopia has provided both survival and immunological benefits to many PLwHAs, and its outcomes are similar to those of ART programs in other countries. The first 6 months of ART is so crucial that program managers and service providers should give due attention during this early course of treatment. Retention of patients in care remains a major challenge, and it is highly variable among health facilities, with high, medium and low retention rates. We, therefore, recommend further studies to analyse and compare the characteristics (structural and process factors) of health facilities with high, medium and low retention rates. Moreover, early initiation of patients on ART should be considered seriously as many patients were started on ART late in the course of their disease: 79% of patients on ART had baseline CD4-cell counts less than 200 cells per micro-liter. Finally, the shift to second-line ART warrants close monitoring to make sure that patients are not taking a failed regimen.

## Competing interests

The authors declare that they have no competing interests.

## Authors' contributions

YA: conceived the study, coordinated and participated in the data collection, conducted the data analysis and interpretation, developed the first draft, and revised subsequent drafts

AK: participated in the data collection and analysis and commented on successive drafts

DT: participated in the data collection and commented on successive drafts.

DH: commented on successive drafts

HK: commented on successive drafts

WE: commented on successive drafts

ML: advised on the data analysis and interpretation and commented on successive drafts

WVD: advised on the conception of the study idea, data analysis and interpretation, commented on successive drafts.

All authors approved the final version for submission.

## Pre-publication history

The pre-publication history for this paper can be accessed here:

http://www.biomedcentral.com/1472-6963/11/81/prepub
